# Development and dosimetric evaluation of a freely deformable ^6^Li‐based neutron shield for boron neutron capture therapy

**DOI:** 10.1002/mp.70319

**Published:** 2026-01-31

**Authors:** Naonori Hu, Ryo Kakino, Akinori Sasaki, Mai Nojiri, Kazuhiko Akita, Syuushi Yoshikawa, Yasushi Kohigashi, Yuki Yoshino, Satoshi Takeno, Teruhito Aihara, Takushi Takata, Hiroki Tanaka, Keiji Nihei, Koji Ono

**Affiliations:** ^1^ Kansai BNCT Medical Center Osaka Medical and Pharmaceutical University Osaka Japan; ^2^ Particle Radiation Oncology Research Center Kyoto University Institute for Integrated Radiation and Nuclear Science Osaka Japan; ^3^ BNCT Joint Clinical Institute Osaka Medical and Pharmaceutical University Osaka Japan; ^4^ Department of Radiology Kyoto Prefectural University of Medicine Kyoto Japan; ^5^ Department of Radiation Oncology Osaka Medical and Pharmaceutical University Hospital Osaka Japan; ^6^ Department of Otorhinolaryngology Osaka Medical and Pharmaceutical University Hospital Osaka Japan

**Keywords:** BNCT, Monte Carlo simulation, neutron shielding

## Abstract

**Background:**

Boron neutron capture therapy (BNCT) enables selective tumor irradiation by exploiting the high‐linear energy transfer particles generated from neutron interactions with ^10^B atoms. BNCT has been approved as an insurance‐covered medical treatment for recurrent head and neck cancer in Japan. Unlike photon radiotherapy, neutrons that come out of the collimator have an angular distribution. Therefore, it is necessary to keep the distance between the collimator and the patient as short as possible. However, for head and neck cancer treatments, patient anatomy often limits proximity to the collimator, creating an unwanted air gap. This ultimately increases the neutron exposure to surrounding healthy tissue.

**Purpose:**

To develop a freely deformable LiF‐polyethylene neutron shield and assess its impact on neutron/gamma attenuation and clinical organ at‐risk sparing in head and neck BNCT.

**Methods:**

A freely deformable neutron shielding device was constructed using polyethylene beads loaded with lithium fluoride encapsulated in a vacuum‐sealed cushion. Neutron and gamma‐ray attenuation were measured in a water phantom under clinical conditions using an accelerator‐based BNCT system (NeuCure®, Kansai BNCT Medical Center). Measurements were compared with a solid LiF‐polyethylene block and validated through Monte Carlo–based simulations in a commercial treatment planning system. Three representative head and neck cases were further simulated to assess clinical dosimetric effects.

**Results:**

The deformable shielding device reduced the thermal neutron flux by approximately 50%, compared with 60% for the solid LiF‐polyethylene block. Simulated head and neck treatments demonstrated significant OAR dose reductions (up to 46.6% in pharyngeal mucosa *D*_50%_) without compromising tumor dose coverage (*D*_80%_ ≥ 20 Gy‐eq). Treatment delivery times were minimally affected (< 2 min difference) across all plans. A 5 mm positional perturbation analysis showed ≤ 0.5 Gy‐eq variation in GTV *D*_min_ and pharyngeal mucosa *D*_50_ and *D*_max_.

**Conclusions:**

The freely deformable LiF‐based neutron shielding device effectively attenuated stray neutron dose while maintaining target coverage in BNCT. Its adaptability and reusability make it a practical adjunct for patient‐specific dose optimization in clinical BNCT applications.

## 1 INTRODUCTION

Boron neutron capture therapy (BNCT) is a type of radiation therapy where a boron compound is administered to the patient, followed by neutron irradiation.^1^ The nuclear reaction between a thermal neutron and a ^10^B atom generates high linear energy transfer (LET) particles that deposit energy over a short range. Therefore, the dose is deposited locally where the reaction took place, minimizing the dose to the surrounding healthy tissue.

Accelerator systems are being developed worldwide for the purpose of generating neutrons for clinical neutron capture therapy.^2^ The world's first accelerator‐based neutron source used in clinical trials was developed by Sumitomo Heavy Industries in collaboration with Kyoto University.^3^, ^4^ This system received approval as a medical device in Japan in March 2020, and insurance coverage for reimbursement for unresectable, locally advanced, or locally recurrent head and neck cancer using this system was approved in June 2020. At Kansai BNCT Medical Center, over 500 patients with recurrent head and neck cancer have been treated with BNCT, with the preliminary results showing high treatment outcomes.^5^

Unlike photon radiotherapy, the collimated neutron field exhibits angular divergence. Therefore, it is important to keep the distance between the collimator and the patient as short as possible to maximize the number of neutrons reaching the tumor and minimize the out‐of‐field dose. However, in head and neck cancer, depending on the tumor location, it is difficult to reduce the air gap as the shoulders prevent bringing the patient close to the collimator. Even with the development of the extended collimator, some air gap remains, particularly when treating the hypopharynx area.^6^ Therefore, a device to reduce unnecessary neutron irradiation that can be fitted for each individual patient treatment is highly sought. ^6^Li is commonly used to shield low‐energy neutrons because its neutron absorption cross section is inversely proportional to the speed of the neutron.^7^ It is preferred over ^10^B in shielding thermal neutrons, as it produces little undesired secondary radiation.^8^, ^9^ This study aims to design and develop a freely deformable neutron shielding device capable of adapting to the unique body contour of each patient, with the objective of minimizing out‐of‐field neutron dose exposure.

## 2 METHOD

### 2.1 Characterization of the deformable neutron shielding device

**FIGURE 1:**
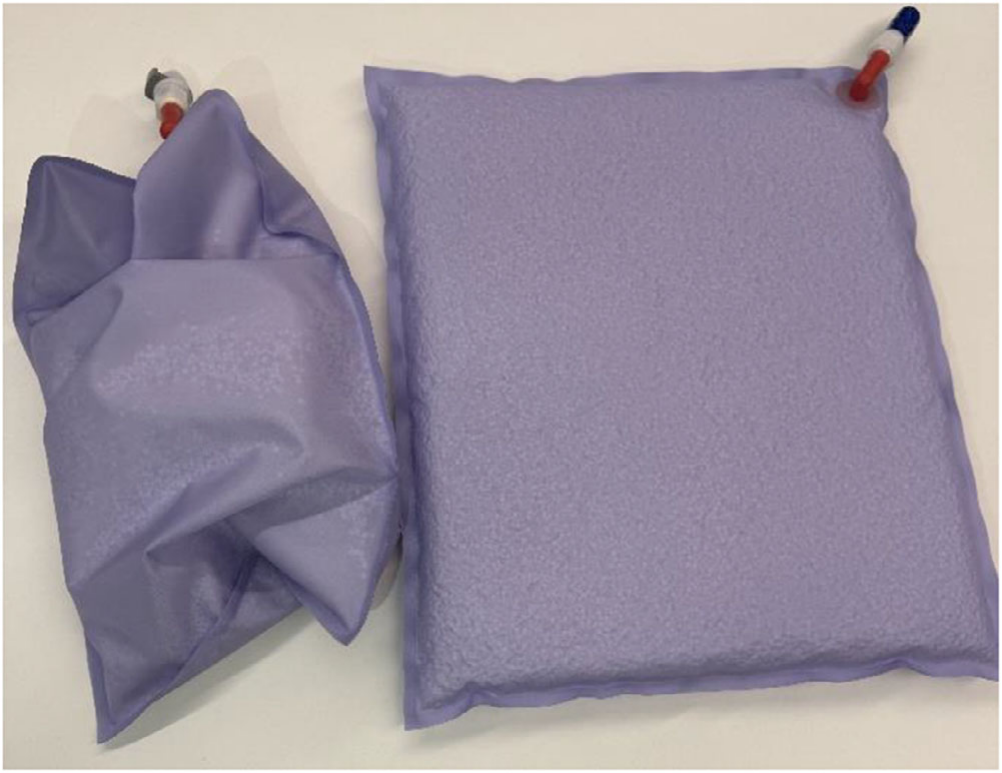
Image of the deformable neutron shielding device. Left: free‐form; right: slab‐form.

**FIGURE 2:**
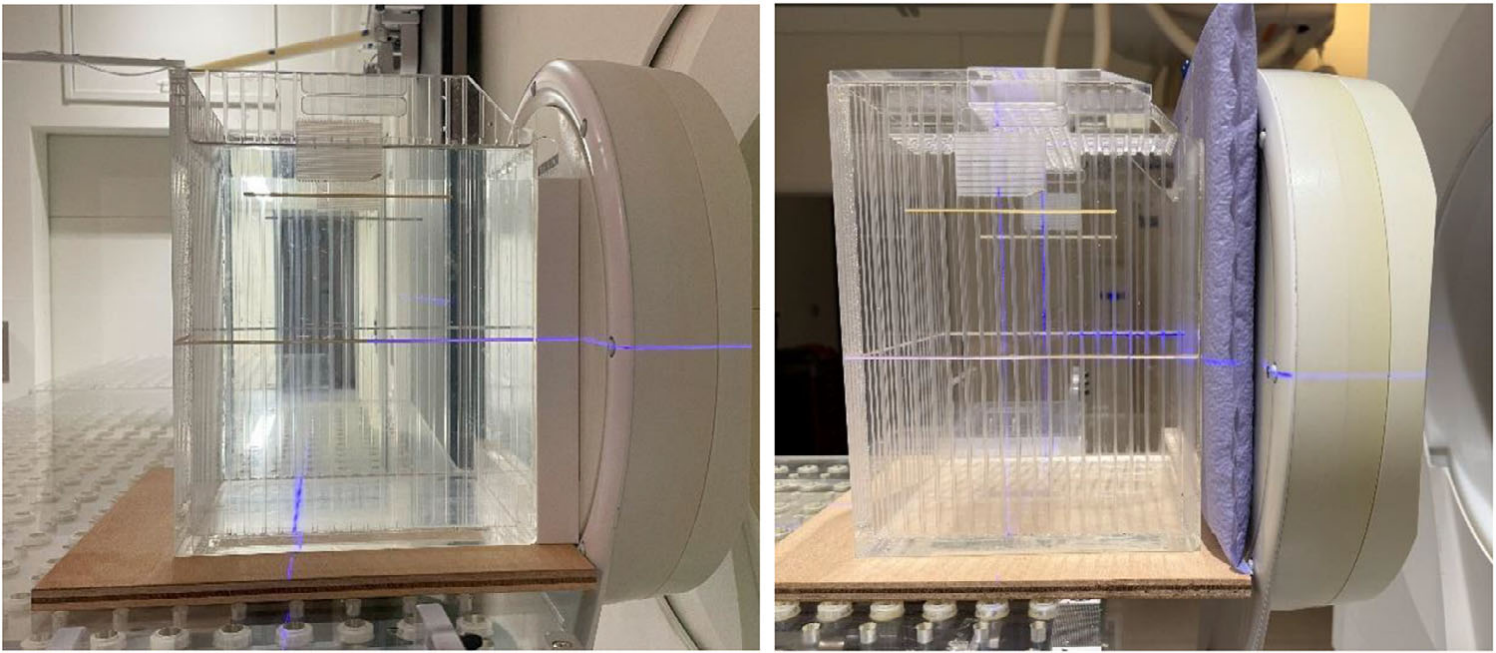
Experimental measurement setup with a solid 25 mm‐thick slab of polyethylene loaded with LiF (left) and the deformable neutron shielding device shaped into a 25 mm‐thick rectangular slab (right).

The deformable neutron shielding device was developed by Engineering System Co., Ltd. (ESCO). Small polyethylene beads loaded with 50 wt% LiF (natural ^6^Li abundance) were placed inside a vacuum‐sealed cushion (Figure 1). A computed tomography (CT) scan was performed to calculate the volume and physical density. To characterize the shielding capability, the device was shaped into a rectangular block (270 mm × 320 mm × 25 mm) and placed in front of a water phantom (Figure 2). Experiments were performed using the accelerator‐based neutron source (NeuCure® BNCT system) installed at the Kansai BNCT Medical Center. A 12 cm diameter circular collimator was used. The thermal neutron and gamma‐ray distribution inside the water phantom was measured using the gold activation method and thermoluminescent dosimeters (TLDs), respectively. A thin gold wire (diameter 0.025 cm × 10 cm in length, with a 99.95% purity, The Nilaco Corporation, Tokyo, Japan) was placed inside the water phantom. The wire was positioned along the central beam axis to measure the depth profile and perpendicular to the beam axis to measure the off‐axis profile. Since gold reacts to both thermal and epithermal neutrons, a cadmium tube cover, manufactured by Shieldwerx (10 cm in length and approximately 1.3 and 2.3 mm for the inner and outer diameter, respectively), was used to calculate the cadmium ratio. After irradiation, the gold wire was cut into small pieces (5 mm in length), and the emitted gamma‐rays were measured using a high‐purity germanium (HPGe) detector (ORTEC ICS‐P4). The reaction rate per unit charge of the gold wire was calculated using the expression below:

$$R=\frac{\lambda N}{\varepsilon \gamma e^{-\lambda T_{C}}\left(1-e^{-\lambda T_{m}}\right)\sum_{i=1}^{n}\left(\frac{Q_{i}}{\Delta t}\left(1-e^{-\lambda \Delta t}\right)e^{-\lambda \left(n-i\right)\Delta t}\right)}$$

where ε is the detection efficiency of the detector for the gamma‐rays emitted from ^198^Au, *γ* is the gamma‐ray emission rate from ^198^Au decay, *λ* is the decay constant of ^198^Au, *T*_c_ is the time from irradiation to the start of the measurement, *T*_m_ is the measurement time, *N* is the peak count due to the detector‐measured gamma‐rays emitted from ^198^Au, and *Q*_i_ is the electric charge irradiated on the target at each interval, Δ*t*.

Gamma‐ray dose measurements were performed using custom‐made TLDs consisting of powdered beryllium oxide encapsulated in quartz glass capsules.^10^ The Panasonic UD‐5120PGL TLD reader was used to measure the signal of the TLD after irradiation. The dosimeters with a ^60^Co gamma‐ray source (414 TBq as of February 2008) at the Kyoto University Institute for Integrated Radiation and Nuclear Science are traceable to a secondary standard ionization chamber certified by the Japan Calibration Service System. Further details of the calibration procedures are described elsewhere.^11^

The results were compared with a solid block of polyethylene loaded with LiF (200 mm × 200 mm × 25 mm, density of 1400 kg/m^3^, same LiF fraction and ^6^Li enrichment as above), purchased from Atom‐Shield. The thermal neutron and gamma‐ray distribution inside the water phantom was measured using the method outlined above.

The experimental setup was modelled using the RayStation treatment planning system (version 2023b), and the simulated results were compared with the experimental measurements. Neutron transport simulation and dose calculations were performed with the NeuCure Dose Engine,^12^ which is a backend computational module built upon the Particle and Heavy Ion Transport code System (PHITS) Monte Carlo code.^13^ The Japanese Evaluated Nuclear Data Library 4.0 (JENDL 4.0) developed by the Japan Atomic Energy Agency (JAEA) was used.^14^ In the TPS, the voxel size was set to 2 mm^3^ with a Monte Carlo uncertainty set to 2%.

### 2.2 Simulation of a clinical head and neck case

**FIGURE 3:**
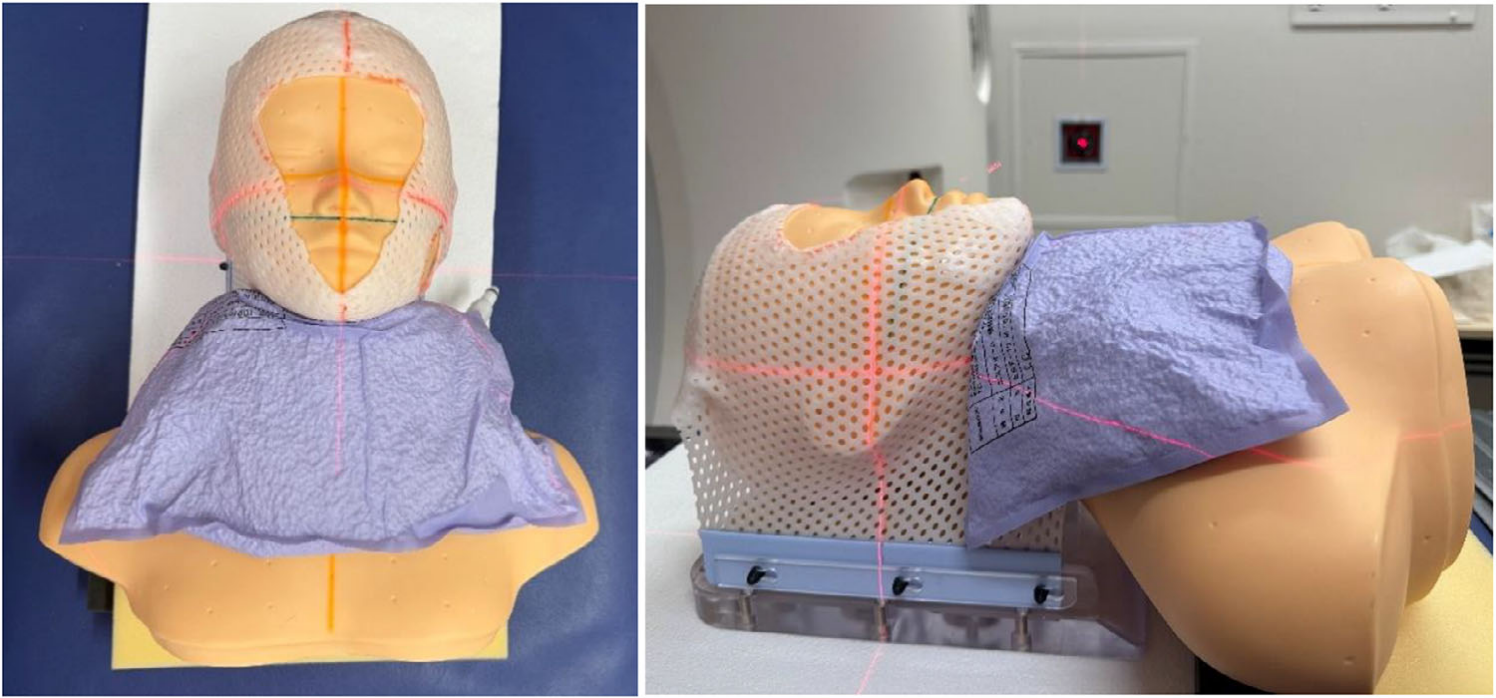
Head and neck phantom (Case 1) with the deformable neutron shielding device placed in front of the larynx region.

Three cases of head and neck cancer treatment were simulated using a head and neck phantom. Case 1: oropharyngeal cancer with an anterior neutron beam; Case 2: right ear canal cancer with a lateral neutron beam; Case 3: oropharyngeal cancer with a 45‐degree angle neutron beam. The deformable shielding device was placed on the phantom in regions where neutron leakage was determined to be significant (Figure 3). A CT scan was performed, and treatment planning and dose calculation were performed with and without the deformable shielding device. The prescription dose was set to *D*_80%_ > 20 Gy‐eq (x‐ray equivalent dose of 20 Gy delivered in a single fraction to at least 80%) of the gross tumor volume (GTV), which has been reported to be the curative dose indicator for recurrent head and neck cancer.^5^, ^15^ The dose delivered to the GTV and organs at risk was calculated and compared with the simulation without the shielding device.

### 2.3 Positioning uncertainty analysis

To evaluate dosimetric sensitivity to potential placement uncertainty of the deformable shielding device, additional dose recalculations were performed by translating the device by 5 mm in each of six directions (left, right, anterior, posterior, superior, and inferior) for Cases 1–3. For each case, the largest deviation from the nominal (no‐shift) calculation was recorded.

## 3 RESULTS

**FIGURE 4:**
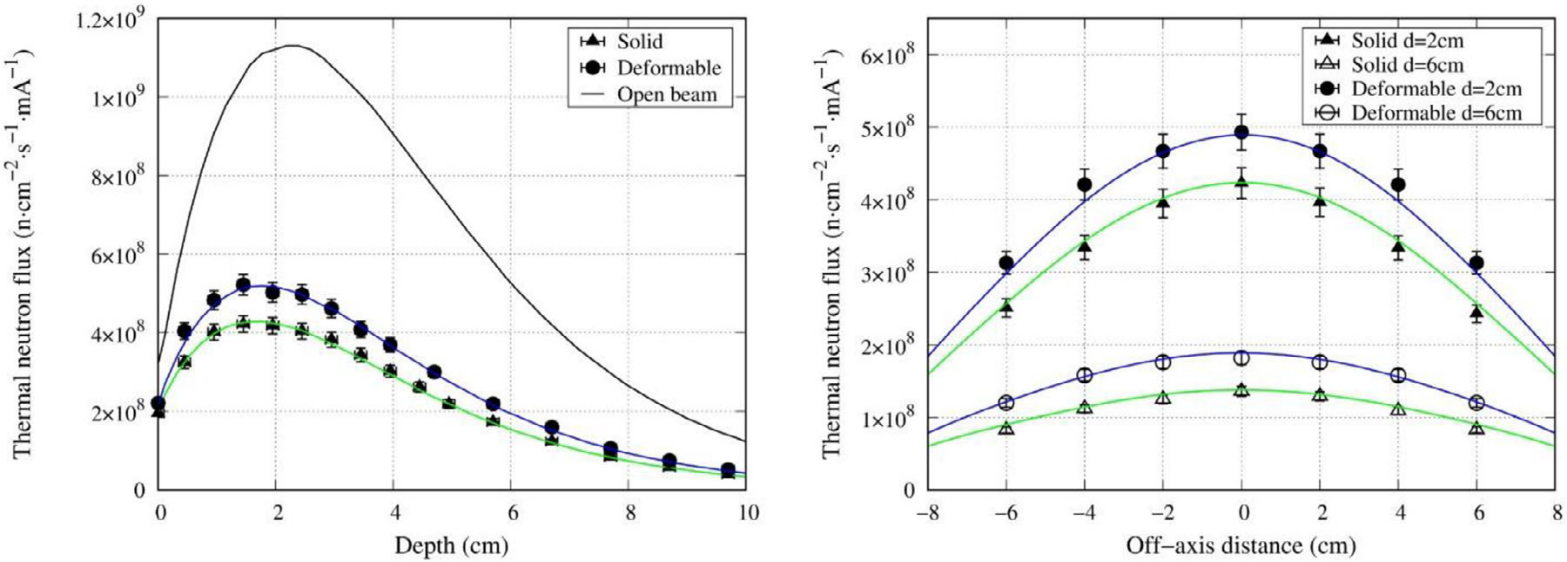
Central axis (left) and off‐axis thermal neutron distribution (right) at depths of 2 and 6 cm. The blue line indicates the slab, the green line indicates the deformable shielding device, and the black line indicates the open beam.

**FIGURE 5:**
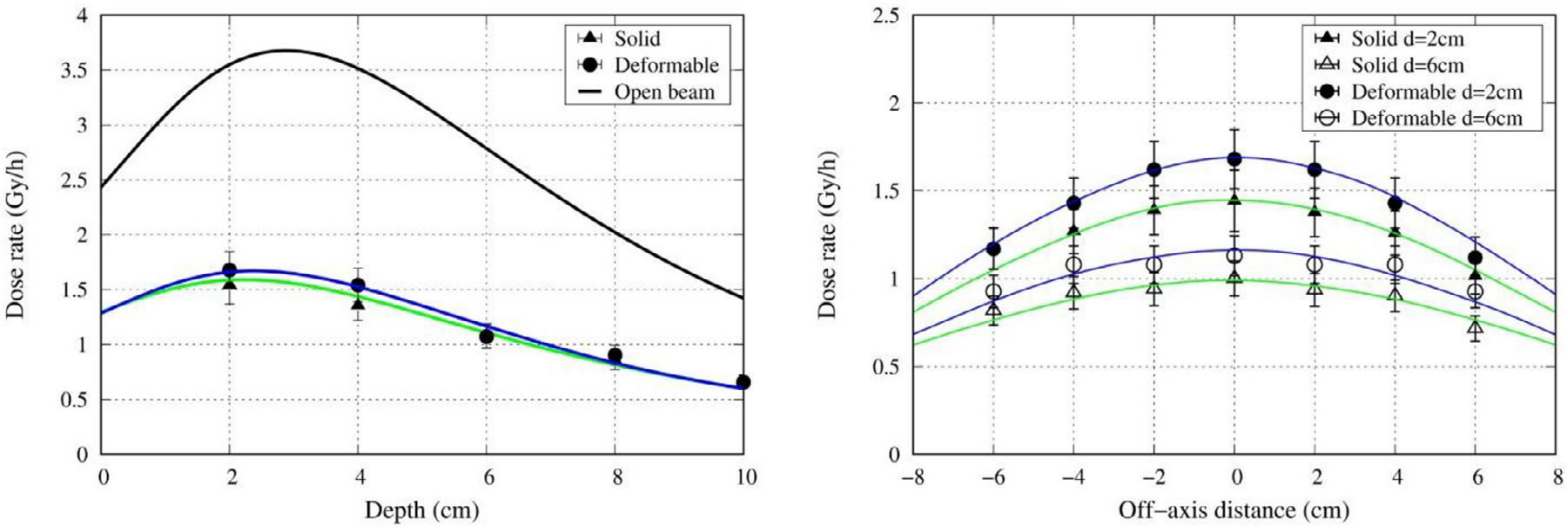
Central axis (left) and off‐axis gamma‐ray dose rate distribution (right) at depths of 2 and 6 cm. The blue line indicates the slab and the green line indicates the deformable shielding device.

The spatial distributions of thermal neutrons along the central axis and off‐axis directions are presented in Figure 4, while those of gamma‐rays are shown in Figure 5. When compared with the open beam condition, the solid 25 mm thick slab of polyethylene loaded with 50% LiF reduced the thermal neutron flux inside the water phantom by approximately 60%, whereas the deformable neutron shielding device reduced the flux by approximately 50%. The relative expanded uncertainty of the gold foil activation method was estimated to be approximately ±5%. The value accounts for the combined effects of several independent components, including the calibration source activity (1.15%), counting statistic (1.0%), measurement repeatability (1.2%), and experimental setup uncertainty (1.0%). The resulting overall uncertainty is consistent with values reported previously for thermal neutron flux determination using comparable methodologies.^16^, ^17^, ^18^ For the TLD measurement, the total uncertainty has been reported to be approximately ±20%.^19^ The simulation results exhibited excellent agreement with experimental measurements (within experimental uncertainty), demonstrating the validity of the computational model.

**FIGURE 6:**
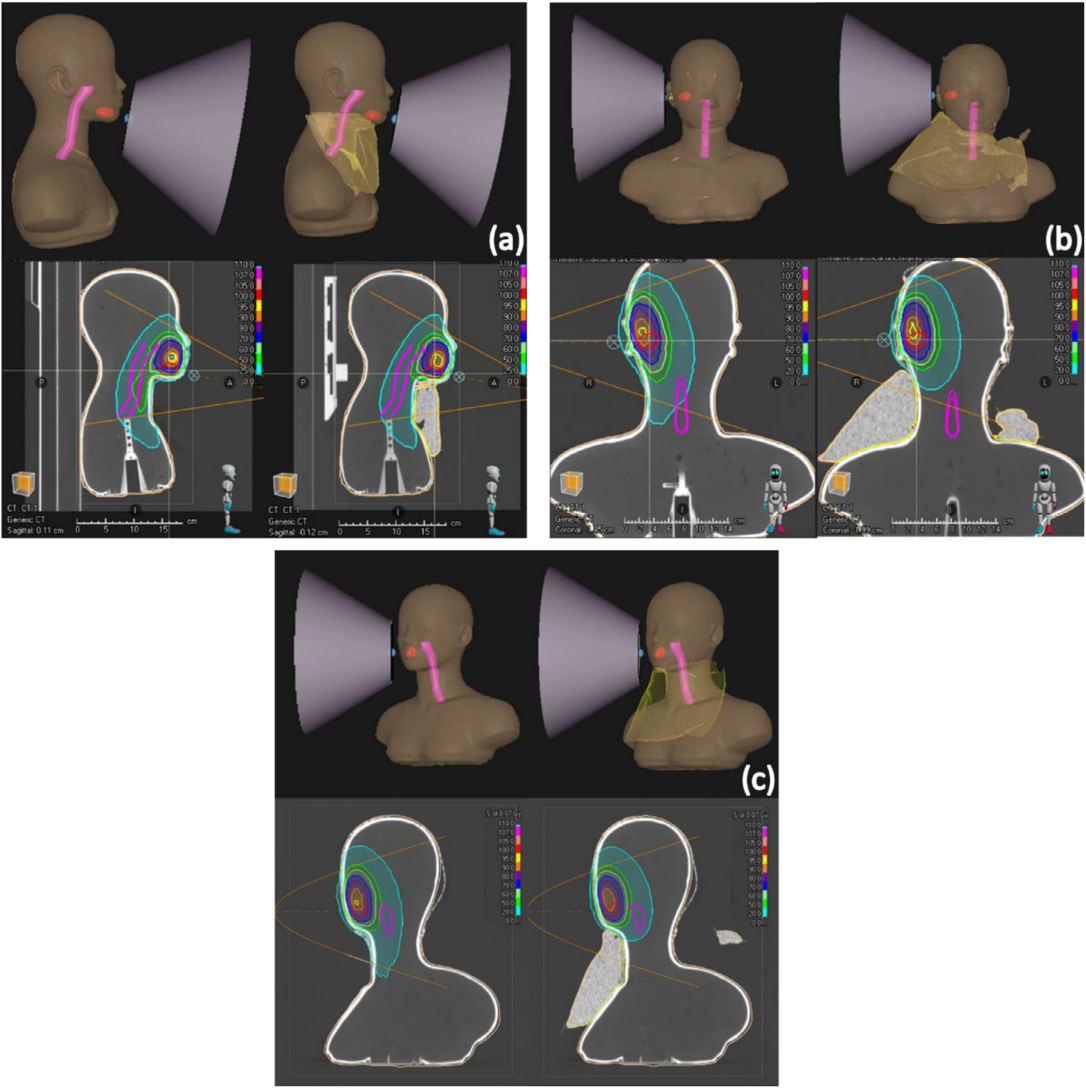
Two‐dimensional distribution of the thermal neutron fluence without (left) and with (right) the deformable neutron shielding device of (a) Case 1, (b) Case 2, and (c) Case 3. The color bar represents the thermal neutron fluence in relative units (%).

The neutron beam arrangement and the two‐dimensional thermal neutron fluence distribution calculated using the RayStation TPS for the three different clinical cases are presented in Figure 6. The results clearly demonstrate that, with the deformable shielding in place, the lateral spread of the thermal neutrons is effectively reduced, particularly near the pharyngeal mucosa (magenta structure), while the thermal neutron fluence at the target region (red structure) is maintained.

**FIGURE 7:**
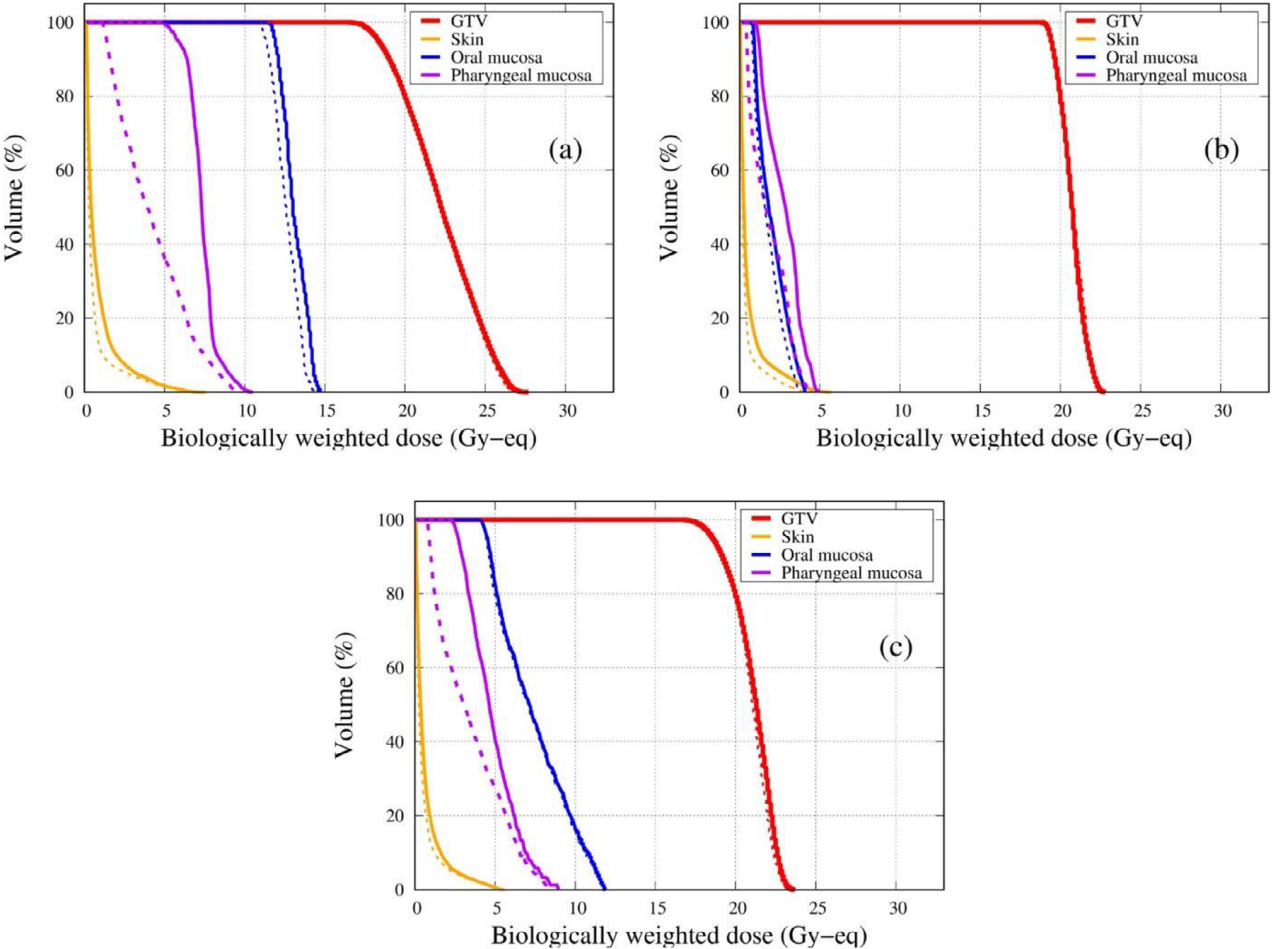
Dose‐volume histogram of (a) Case 1, (b) Case 2, and (c) Case 3. A solid line indicates calculation with no shielding; a dashed line indicates deformable shielding.

The dose‐volume histogram of the three different clinical cases is shown in Figure 7 and summarized in Table 1. The BNCT dose calculation method is summarized in the Appendix. In all cases, no significant change in the GTV dose was observed, while the dose to organs at risk (particularly the pharyngeal mucosa) was notably reduced. The treatment times required to deliver the prescribed dose (*D*_80%_ > 20 Gy‐eq) for Case 1, Case 2, and Case 3 were 35.9/34.0, 21.3/21.4, and 23.5/22.8 min for the without/with shielding conditions, respectively.

**TABLE 1:** Summary of simulated head and neck BNCT cases with and without the deformable neutron shield.

Case	Beam orientation	GTV *D*_min_ (Gy‐eq)	Pharyngeal mucosa *D*_50_ (Gy‐eq)	Pharyngeal mucosa *D*_max_ (Gy‐eq)	Treatment time (min)	Change in OAR *D*_50_ (%)
1	Anterior	16.3/16.6	7.3/3.9	10.5/9.8	35.9/34.0	46.6
2	Lateral	18.7/18.7	2.9/1.6	4.9/4.5	21.3/21.4	44.8
3	Oblique 45°	16.6/16.8	4.6/3.1	9.0/8.6	23.5/22.8	32.6

*Note*: Values are given without/with shielding.

Treatment time corresponds to the beam‐on duration required to achieve prescribed GTV *D*_80_ ≥ 20 Gy‐Eq.

A positioning uncertainty analysis was performed by recalculating dose after shifting the deformable shielding device by 5 mm in each of six directions (left, right, anterior, posterior, superior, and inferior). The worst‐case (largest deviation from the nominal no‐shift plan) values were Case 1, GTV *D*_min_ 17.1 Gy‐eq, pharyngeal mucosa *D*_50_ 4.2 Gy‐eq, and pharyngeal mucosa *D*_max_ 10.3 Gy‐eq; Case 2, GTV D_min_ 18.8 Gy‐eq, pharyngeal mucosa *D*_50_ 1.4 Gy‐eq, and pharyngeal mucosa *D*_max_ 4.3 Gy‐eq; and Case 3, GTV *D*_min_ 16.6 Gy‐eq, pharyngeal mucosa *D*_50_ 2.9 Gy‐eq, and pharyngeal mucosa *D*_max_ 8.8 Gy‐eq. Compared with the nominal shielded plans, the maximum absolute deviation was ≤ 0.5 Gy‐eq for these endpoints.

## 4 DISCUSSION

The neutron and gamma‐ray distribution inside a water phantom was experimentally evaluated for the newly developed deformable neutron shielding device using the accelerator‐based neutron source at the Kansai BNCT Medical Center. Compared with a solid block of the same material (polyethylene loaded with 50% LiF), the thermal neutron shielding ability was 16.9% less. This may be due to the small air gaps that existed within the device. Regardless, the deformable neutron shielding device was able to reduce the thermal neutron flux by 50% when compared with the open beam. The deformable neutron shielding device had a different dimension than the solid slab (270 × 320 mm^2^ vs. 200 × 200 mm^2^), due to the limitation in the manufacturing process. Because the two shields differed in lateral dimensions (270 × 320 mm^2^ for the deformable device versus 200 × 200 mm^2^ for the solid block), the potential influence of lateral size on neutron scatter and attenuation warranted further evaluation. To isolate this factor, we performed additional Monte Carlo simulations of the deformable shielding device using both the original geometry (270 × 320 mm^2^) and a reduced geometry matching the solid block (200 × 200 mm^2^). The resulting central‐axis thermal neutron flux depth curves were nearly identical, with a peak percentage difference of < 1% between the two lateral sizes. This indicates that, for the central‐axis depth profile used to quantify the peak attenuation in this study, the lateral size difference has a negligible effect. Therefore, the observed 16.9% reduction in attenuation relative to the solid block remains valid at the maximum peak and is more plausibly attributable to the internal structure of the deformable device.

The simulation results of the three head and neck cases all showed a decreased dose to the pharyngeal mucosa with the use of the deformable neutron shielding device. The two‐dimensional thermal neutron flux distribution clearly showed the reduction in the thermal neutron fluence behind the shielding device. Out of the three cases, the dose reduction was the largest with the oropharyngeal case with an anterior beam orientation, where the D_50%_ of the pharyngeal mucosa dose decreased by 46.6%. The application of this shielding device has the potential to mitigate radiation‐induced side effects, such as mucositis. In addition to reducing the dose to organs at risk, it also reduces the out‐of‐field neutron dose,^20^ thereby contributing to an overall improvement in the safety and efficacy of BNCT treatments.

Although this study focused on head and neck BNCT, the concept of a patient‐conformal, freely deformable neutron shield may be applicable to other anatomical sites where unavoidable collimator‐to‐patient air gaps or nearby organs at risk lead to elevated out‐of‐field neutron exposure. For example, in intracranial BNCT (e.g., glioblastoma), conformal shielding placed over superficial regions may help reduce dose to the scalp or ocular structures depending on the beam arrangement. More broadly, the device may be used to locally attenuate stray thermal neutrons outside the target region without increasing the collimator‐to‐patient distance, provided that the shield does not intersect the direct beam path.

For clinical implementation, the device can be molded after patient immobilization to conform to the patient surface in regions of elevated stray neutron fluence adjacent to organs at risk while avoiding the direct beam path. The cushion is then vacuum‐sealed to maintain its shape, and a planning CT can be acquired with the device in place so that it is explicitly represented in the dose calculation; no special modification to the CT protocol is required other than ensuring the device is fully included within the scan range. Although the additional setup time was not quantified in this study, the workflow is analogous to that of commonly used vacuum cushions and is expected to require minimal additional time. In addition, the 5 mm shift sensitivity analysis demonstrated changes of ≤ 0.5 Gy‐eq in GTV *D*_min_ and pharyngeal mucosa *D*_50_/*D*_max_, suggesting that the dosimetric benefit is robust to small placement variations. Nevertheless, the reproducibility of device placement should be evaluated, and image‐guided systems (e.g., cone‐beam CT) or surface‐guided systems may assist in ensuring consistent positioning.

## 5 CONCLUSIONS

A freely deformable, patient‐specific neutron shielding device was developed and evaluated under a clinical BNCT treatment system. This device can be readily molded to conform to the unique surface contour of each patient and can be easily reused for subsequent treatments. The application of this device has the potential to reduce neutron exposure to surrounding organs at risk while maintaining the prescribed therapeutic dose to the target area.

## CONFLICT OF INTEREST STATEMENT

The authors declare no conflicts of interest.

## Data Availability

The data that support the findings of this study are available from the corresponding author upon reasonable request.

## BNCT DOSE CALCULATION

In BNCT, the total absorbed dose delivered to the patient is expressed as the sum of several physical dose components. The biologically weighted dose (Gy‐eq) represents the sum of these components, each multiplied by its corresponding compound biological effectiveness (CBE) or relative biological effectiveness (RBE):

$$\begin{array}{cc} D_{T} & =CBE\times^{10}Bconcentration\times D_{B}+RBE_{H}\times D_{H}\\ & \;+RBE_{N}\times D_{N}+RBE_{G}\times D_{G} \end{array}$$

where *D_T_* is the total biologically weighted dose. The boron dose (*D_B_*) arises from the ^10^B(n,α)^7^Li reaction, in which the emitted α‐particle and recoiling ^7^Li ion deposit approximately 2.33 MeV locally. The hydrogen dose (*D_H_*) results from recoil protons generated by epithermal and fast neutrons interacting with hydrogen atoms. The nitrogen dose (*D_N_*) results from the ^14^N(n,p)^14^C reaction, producing protons. The gamma‐ray dose (*D*_*G*_) is due to both the primary gamma‐rays in the incident beam and those generated within tissue, the ^1^H(n,γ)^2^H reaction. The parameters *RBE_H_*, *RBE_N_*, and *RBE_G_* denote the RBE for hydrogen, nitrogen, and gamma‐ray components, respectively. In this study, *RBE_H_* was set to 2.4, *RBE_N_* was set to 2.9, and *RBE_G_* was set to 1.0. The CBE accounts for microscopic boron distribution and varies with both the boron compound and the tissue type being evaluated. In this study, the CBE for tumor, mucosa, skin, and all other tissue types were set to 3.8, 4.9, 2.5, and 1.34, respectively. The ^10^B concentration for tumor and all other tissue was set to 62.5 and 25 ppm, respectively. Further details of the dose calculation procedure can be found elsewhere.^12^
